# Linear-Structured-Light Measurement System Based on Scheimpflug Camera Thick-Lens Imaging

**DOI:** 10.3390/s24165124

**Published:** 2024-08-07

**Authors:** Dongyu Guo, Jiwen Cui, Yuhang Wu

**Affiliations:** Center of Ultra-Precision Optoelectronic Instrument Engineering, Harbin Institute of Technology, Harbin 150080, China; 19b901005@stu.hit.edu.cn (D.G.); 22b901025@stu.hit.edu.cn (Y.W.)

**Keywords:** measurement field, sensor, structured light, Scheimpflug camera, measurement model

## Abstract

A thick-lens, structured-light measurement model is introduced to overcome the oversights in traditional models, which often disregard the impact of lens thickness. This oversight can lead to inaccuracies in Scheimpflug camera calculations, causing systematic errors and diminished measurement precision. By geometrical optics, the model treats the camera as a thick lens, factoring in the locations of its principal points and the spatial shifts due to image plane tilting. The model deduces the positional relationship of the thick lens with a tilted optical axis and establishes a linear-structured-light measurement model. Simulations confirm that the model can precisely calculate the 3D coordinates of subjects from image light strip data, markedly reducing systematic errors across the measurement spectrum. Moreover, experimental results suggest that the refined sensor model offers enhanced accuracy and lower standard deviation.

## 1. Introduction

High-precision curved parts are widely used in aerospace, transport, and medical devices. If the surface morphology of these parts is abnormal, it may lead to failure [1]. High-precision 3D surface measurement technology is an important guarantee of machining accuracy. However, due to the complexity of the surfaces of these parts, commonly used machines such as coordinate measuring machines, articulated arm measuring machines, and binocular vision systems are unable to meet the demand for high-precision surface topography measurement [2,3,4,5].

Structured-light measurement technology, with its high efficiency and simple structure, is widely used for surface measurements in reverse engineering, industrial inspection, quality control, identification, and positioning [6,7,8,9]. It mainly consists of a laser light source and a camera. The laser light source projects a light beam onto the surface of the measurement object, forming a light band that is captured by the camera. From the geometric distortion of the light band, the 3D coordinates of the surface are calculated.

The camera is a core component of a structured-light measurement system and is responsible for sensing signals and obtaining the 3D coordinates of the object to be measured based on the camera model and its parameters. In the case of measuring curved parts, the undulation of the object surface often distorts the light bar beyond the depth of field of the camera. To address the depth of field limitation, researchers often choose a Scheimfplug camera with a tilted optical axis. The camera is always assumed as a pinhole, which is a significant assumption to use under vertical–optical axis conditions because the object and image have no coordinate changes along the optical axis but do not work well with Scheimfplug cameras. Due to the tilted optical axis of the Scheimfplug camera, both the object and image have a significant coordinate shift in the optical axis direction. The small-aperture approximation is not sufficient to accommodate this shift, resulting in an inability to accurately analyze the relationship between the object and image with the Scheimfplug camera. The linear-structured-light measurement model established on this basis is inaccurate, bringing systematic errors and affecting the measurement accuracy of the linear-structured-light sensor. Therefore, it is necessary to remodel the structured-light measurement system of the Scheimfplug camera with a large aperture [10,11]. 

To address the inaccuracy of the line-structured-light measurement model applied to the Scheimfplug camera, some researchers continued to insist on using the traditional small-hole assumption but could only maintain high accuracy at small angles below 6° [12]. In 2001, Grossberg came up with a general camera model based on focal dispersion surfaces for a variety of special cameras, including Scheimfplug cameras, camera arrays, etc. However, due to the need to trace multiple rays, it was not easy to obtain the focal dispersion plane in experiments, which led to difficulties in solving this model [13]. In 2013, Antonin Miks analyzed the properties of aberrations in the Scheimfplug camera in line-structured-light measurements, but only a computational example was given and practical validation was lacking [14]. In 2016, the Southern Methodist University raised a pupil-centered ray-tracing model, which was complex, cumbersome, and lacked experimental validation [15]. In 2017, Steger presented a new camera model based on the relationship between projected camera matrices, but the accuracy was too low [16]. Subsequently, he used a scanning camera model considering lens aberration, but it could only be applied to the telecenter [17,18]. In 2019, Yin X Q proposed an aberration-based model, but it was difficult to be applied to the reconstruction of 3D measurements [19]. In 2021, Zhang Y established a transformation model from the image coordinate system to the coordinate system of the measurement object through the spatial projection relation [20]. In the same year, Alvarez H proposed a multi-camera sensing model but failed to achieve high accuracy [21]. In 2022, Hu Y proposed a simplified camera model, but only one-dimensional detection was performed [22,23]. 

All the studies mentioned have been categorized as either modifications of the traditional small-aperture model or as overly complex, hindering their practical application in line-structured-light measurements. The systematic errors introduced to the camera model led to large errors when solving for the 3D coordinates of the object from image light bands, which reduced the accuracy of the structured-light sensors and did not allow for current high-precision measurements [19,24,25]. Therefore, there is a need to investigate an accurate and systematic structured-light measurement model, which should go beyond the small-aperture model while maintaining good practicality.

In this paper, a structured-light measurement model was derived from the Scheimfplug camera thick-lens imaging principle to achieve accurate measurement. The imaging matrix was established according to the ideal optical imaging principle, the spatial position of the image point was deduced according to the optical axis tilt condition, and the relationship between the pixel coordinates and the image plane coordinates was derived, which was combined with the optical plane equation to obtain an accurate and practical linear-structured-light measurement model. It is shown through simulation and experiment that the proposed model compensates more for the magnification and imaging position than the conventional small-hole model, reduces the systematic error of the model, and has higher accuracy.

The rest of the paper is organized as follows: In Section 2, a structured-light measurement model is built based on the Scheimpflug imaging principle for thick lenses. Section 3 establishes the measurement system, simulates the two models, and conducts system calibration experiments, measurement block verification tests, and bearing measurement experiments to verify the feasibility of the models and their advantages over conventional models. The fourth part is the conclusion.

## 2. Scheimfplug Imaging System Model

### 2.1. Linear-Structured-Light Measurement Model

**Figure 1:**
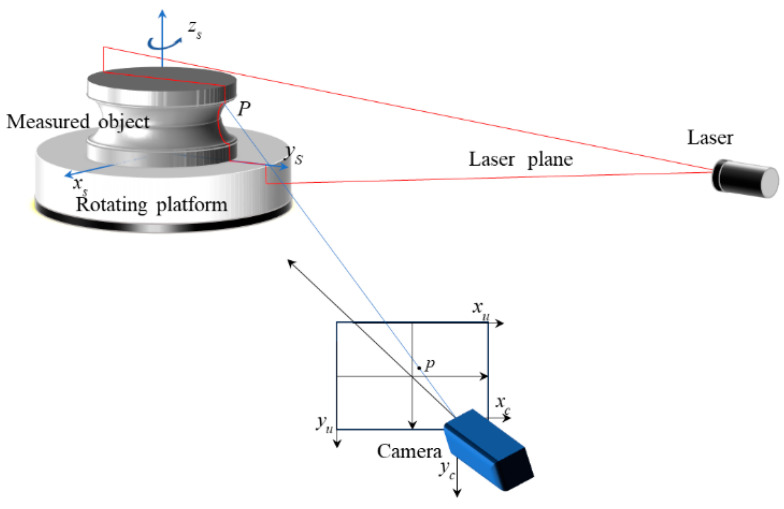
Linear-structured-light measurement model.

The linear-structured-light measurement model is shown in Figure 1. A laser plane is projected onto the measured surface by the laser source to create a light strip. Point P is a point on the light strip, photographed with a camera and imaged on the CCD (charge-coupled device). The corresponding image point is the point p. The upper-left corner of the image is taken as the origin of the pixel coordinate system, $O\text{-}x_{u}y_{u}$. The origin of the camera coordinate system $O\text{-}x_{c}y_{c}z_{c}$ is the optical center, and the optical axis is the $z_{c}$-axis. Then, the relationship between point p and point P is (1).

(1) $${\left(\begin{array}{ccc} x_{c} & y_{c} & z_{c} \end{array}\right)}^{T}=\lambda K{\left(\begin{array}{ccc} x_{u} & y_{u} & 1 \end{array}\right)}^{T}$$

${\left(\begin{array}{ccc} x_{c} & y_{c} & z_{c} \end{array}\right)}^{T}$ are the 3D coordinates of point P in the camera coordinate system. λ is an indefinite factor and K is the imaging matrix. ${\left(\begin{array}{ccc} x_{u} & y_{u} & 1 \end{array}\right)}^{T}$ are the plane coordinates of point p in the pixel coordinate system. Model (1) shows that a linear relationship exists between the coordinates of the object point and the corresponding image point. This relationship is fundamental to understanding how the structured-light measurement model correlates to the physical position of a point on the measured surface with its representation on the camera’s image sensor. The equation encapsulates the mathematical foundation that allows for the accurate conversion of pixel coordinates to real-world coordinates within the context of the structured-light system.

Because of an unknown trimming factor λ in the formula, only one ray passing through P can be obtained, so a constraint condition is needed. In the linear-structured-light measurement system, P is substituted to the laser plane equation to determine the 3D coordinates, as presented in (2):

(2) $$Ax_{c}+By_{c}+Cz_{c}=1$$

A, B, and C in (2) are the coefficients of $x_{c}$, $y_{c}$, and $z_{c}$ of the laser plane in the camera coordinate system. The three coefficients are represented by a vector, $\left(\begin{array}{ccc} A & B & C \end{array}\right)$. By substituting the plane equation into (1), the solution of the indefinite factor *λ* is $1/\left(\left(\begin{array}{ccc} A & B & C \end{array}\right)K{\left(\begin{array}{ccc} x_{u} & y_{u} & 1 \end{array}\right)}^{T}\right)$.

Substituting λ into (1) gives the final measurement model in (3):

(3) $${\left(\begin{array}{ccc} x_{c} & y_{c} & z_{c} \end{array}\right)}^{T}=\frac{K{\left(\begin{array}{ccc} x_{u} & y_{u} & 1 \end{array}\right)}^{T}}{\left(\begin{array}{ccc} A & B & C \end{array}\right)K{\left(\begin{array}{ccc} x_{u} & y_{u} & 1 \end{array}\right)}^{T}}$$

Model (3) presents the coordinates of a spatial curve truncated by the laser plane. According to (3), in the case of a single measurement, the relationship between the object point and the corresponding image point is a rational function, rather than a simple linear one.

For a complete measurement, additional movements are required to make the laser scan the entire contour. Transforming the solved coordinate points from the camera coordinate system to the measurement coordinate system is a rigid body transformation, as is each scanning motion. Therefore, the line-structured-light measurement model is (4).

(4) $${\left(\begin{array}{ccc} x_{m} & y_{m} & z_{m} \end{array}\right)}^{T}=R_{i}\left(R_{c2m}{\left(\begin{array}{ccc} x_{c} & y_{c} & z_{c} \end{array}\right)}^{T}+{\vec{t}}_{c2m}\right)+{\vec{t}}_{i}$$

$R_{c2m},{\vec{t}}_{c2m}$ is the rotation matrix and translation vector from the camera coordinate system to the measurement coordinate system. $R_{i},{\vec{t}}_{i}$ is the rotation matrix and translation vector of the transformed object position and initial position funding for the ith measurement. It can be seen from (4) that the measurement results are also affected by scanning motion. 

According to the model, the main influencing factors of the linear-structured-light measurement system are (1) light stripe extraction, (2) camera model accuracy, (3) optical plane calibration accuracy, and (4) scanning motion accuracy. The main problem to be solved in this paper is the correction of the camera model.

### 2.2. Scheimfplug Camera Model

The camera model is a central part of structured-light measurement. All information is collected with a camera, and the optical plane calibration is also transformed into 3D coordinates utilizing this model. Currently, the camera is always assumed to have a small aperture and is described as an imaging matrix based on the triangle similarity principle, namely the pinhole model [26]. The small-aperture assumption is an accurate one in the case of weak $z_{c}$ variations.

However, since the Scheimfplug camera is a shifted-axis camera, the object image is offset along the optical axis, causing a macroscopic change in $z_{c}$. This change is beyond the pinhole model, making the accuracy decrease rapidly as the change in $z_{c}$ increases. This means that K in (3) introduces a systematic error, and the solution for the object–image relationship is inaccurate, affecting the measurement accuracy. Therefore, in this section, a Scheimfplug model with lens thickness is established to replace the pinhole model based on the ideal optical imaging principle rather than the small-aperture assumption.

Supposedly, the pixel coordinate is established in Figure 1 with the pixel point $p_{u}={\left(\begin{array}{ccc} x_{u} & y_{u} & 1 \end{array}\right)}^{T}$, whose origin is the intersection of the optical axis and CCD plane and the coordinate axes are the same as the CCD. The image coordinates are parallel to the pixel coordinates and the untilted image coordinates of point $p_{u}$ are presented in (5):

(5) $$\left(\begin{array}{c} x_{im}\\ y_{im}\\ 1 \end{array}\right)=\left(\begin{array}{ccc} d_{x} & 0 & -d_{x}x_{0}\\ 0 & d_{y} & -d_{y}y_{0}\\ 0 & 0 & 1 \end{array}\right)\left(\begin{array}{c} x_{u}\\ y_{u}\\ 1 \end{array}\right)$$

$d_{x},d_{y}$ are the pixel size of the $x_{u}$, $y_{u}$ direction. $x_{0},y_{0}$ are the $x_{u}$, $y_{u}$ coordinates of the intersection point between the optical axis and the image plane. The units of image points are converted from pixels to millimeters in (5).

In terms of camera coordinates, the untilted coordinate of point $p_{u}$ is presented in (6).

(6) $$\left(\begin{array}{c} x_{im}\\ y_{im}\\ z_{0}\\ 1 \end{array}\right)=\left(\begin{array}{ccc} 1 & 0 & 0\\ 0 & 1 & 0\\ 0 & 0 & z_{0}\\ 0 & 0 & 1 \end{array}\right)\left(\begin{array}{c} x_{im}\\ y_{im}\\ 1 \end{array}\right)$$

$z_{0}$ is the z-coordinate of point $p_{u}$ in the camera coordinate system. The position of the image plane relative to the camera is fixed in (6).

In terms of camera coordinates, point ${\left(\begin{array}{ccc} 0 & 0 & z_{0} \end{array}\right)}^{T}$ is the intersection of the optical axis and the CCD plane. Considering the tilt angle, the relationship between the camera coordinate point and its corresponding image point is confirmed in (7).

(7) $$\left(\begin{array}{c} {x_{c}}^{\prime }\\ {y_{c}}^{\prime }\\ {z_{c}}^{\prime }\\ 1 \end{array}\right)=\left(\begin{array}{cccc} \cos\theta & -\sin\varphi \sin\theta & \cos\varphi \sin\theta & -z_{0}\cos\varphi \sin\theta \\ 0 & \cos\varphi & -\sin\varphi & z_{0}\sin\varphi \\ \sin\theta & \sin\varphi \cos\theta & \cos\varphi \cos\theta & z_{0}-z_{0}\cos\varphi \cos\theta \\ 0 & 0 & 0 & 1 \end{array}\right)\left(\begin{array}{c} x_{im}\\ y_{im}\\ z_{0}\\ 1 \end{array}\right)$$

*φ*, *θ* is the camera image plane 2D tilt angle. After the tilt of (7), the image plane and lens constitute the basic structure of the Scheimfplug camera.

Associating (6) and (7) and simplifying them, the relationship between the camera coordinate point and its corresponding CCD point is in (8):

(8) $${\left(\begin{array}{cccc} {x_{c}}^{\prime } & {y_{c}}^{\prime } & {z_{c}}^{\prime } & 1 \end{array}\right)}^{T}=A{\left(\begin{array}{ccc} x_{u} & y_{u} & 1 \end{array}\right)}^{T}$$

Mark matrix $A=\left(\begin{array}{ccc} \cos\theta d_{x} & -\sin\varphi \sin\theta d_{y} & -\cos\theta d_{x}x_{0}+\sin\varphi \sin\theta d_{y}y_{0}\\ 0 & \cos\varphi d_{y} & -\cos\varphi d_{y}y_{0}\\ \sin\theta d_{x} & \sin\varphi \cos\theta d_{y} & z_{0}-\sin\theta d_{x}x_{0}-\sin\varphi \cos\theta d_{y}y_{0}\\ 0 & 0 & 1 \end{array}\right)$. 

The relationship between the coordinate points of an image and the corresponding image points in the Scheimfplug camera is described in (8).

### 2.3. Thick-Lens Imaging Combined Scheimfplug Camera Model

**Figure 2:**
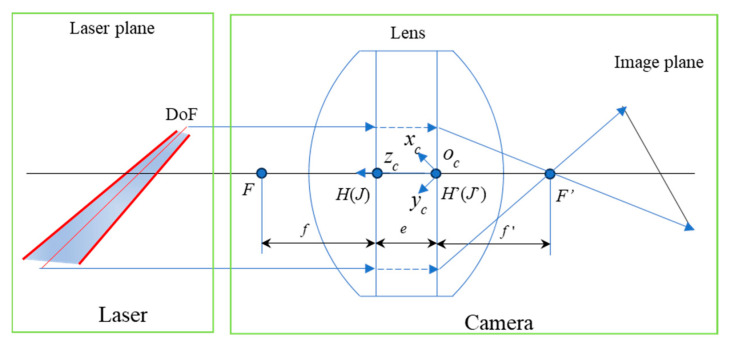
Scheimpflug imaging system model.

An ideal optical system has three sets of cardinal points, i.e., the focal point, the principal point, and the node, where the principal point and the node usually coincide. The two principal points of the object and image planes are assumed to coincide as the center of light and are designated as the origin of the system in the pinhole model. Therefore, according to the triangular similarity property, the ratio of the z-coordinate of the image point to the z-coordinate of the object point is the magnification. In practice, an interval exists between the two principal points, which is important in a thick-lens model, while the interval is negligible in the small-aperture assumption, as in Figure 2. 

The camera coordinate system is established with the main point of the image side as the origin, as in Figure 2. The optical axis is directed from the image side towards the object side in the positive direction of the z-axis, and the x-axis and y-axis are aligned parallel to the x-axis and y-axis of the image coordinate system that is not tilted. It is assumed that the coordinates of the principal points on the object side are ${\left(\begin{array}{ccc} 0 & 0 & e \end{array}\right)}^{T}$, the focal length is f, the coordinates of the focus on the object side are ${\left(\begin{array}{ccc} 0 & 0 & e+f \end{array}\right)}^{T}$, the coordinates of the focus on the image side are ${\left(\begin{array}{ccc} 0 & 0 & -f \end{array}\right)}^{T}$, and the z-coordinates of the object point and image point are $z_{c}$ and ${z_{c}}^{\prime }$, respectively.

According to Newton’s formula $xx^{\prime }=ff^{\prime }$, we obtain (9):

(9) $$\left\{\begin{array}{l} {x_{c}}^{\prime }=\beta_{m}x_{c}\\ {y_{c}}^{\prime }=\beta_{m}y_{c}\\ \left({z_{c}}^{\prime }-f^{\prime }\right)\left(z_{c}-e-f\right)=ff^{\prime } \end{array}\right.$$

$x,x^{\prime }$ is the object distance and image distance, where $x=z_{c}-e-f,x^{\prime }={z_{c}}^{\prime }-f^{\prime }$. $f,f^{\prime }$ is the focal length of object and focal length of image; in general, $f=-f^{\prime }$. $\beta_{m}$ is the vertical magnification, $\beta_{m}=-\frac{f}{x}=-\frac{x^{\prime }}{f^{\prime }}$. In the thick-lens hypothesis, the image point coordinates of the camera and the corresponding object point coordinates are governed by the ideal optical imaging principle, known from (9).

Combining $z_{c}$, we obtain (10).

(10) $$\left\{\begin{array}{c} x_{c}=\frac{f}{{z_{c}}^{\prime }+f}{x_{c}}^{\prime }\\ y_{c}=\frac{f}{{z_{c}}^{\prime }+f}{y_{c}}^{\prime }\\ z_{c}=\frac{{z_{c}}^{\prime }\left(e+f\right)+ef}{{z_{c}}^{\prime }+f} \end{array}\right.$$

The above Equation (10) indicates that in the thick-lens hypothesis, the key parameter between the coordinate of the image point and the coordinate of the object point is ${z_{c}}^{\prime }$, and the relationship between ${z_{c}}^{\prime }$ and $z_{c}$ is the inverse proportional function of the offset.

In matrix form, it is (11):

(11) $$P_{c}=\left(\begin{array}{c} x_{c}\\ y_{c}\\ z_{c} \end{array}\right)=\lambda \left(\begin{array}{cccc} f & 0 & 0 & 0\\ 0 & f & 0 & 0\\ 0 & 0 & e+f & ef \end{array}\right)\left(\begin{array}{c} {x_{c}}^{\prime }\\ {y_{c}}^{\prime }\\ \begin{array}{c} {z_{c}}^{\prime }\\ 1 \end{array} \end{array}\right)=\lambda B{P_{c}}^{\prime }$$

The same denominator in (10) is presented as the coefficient of the matrix in (11). The image relation of the camera is interpreted in (11) as a matrix with undetermined coefficients, which are related to the ${z_{c}}^{\prime }$ coordinates. Combining (8) and (11), the Scheimfplug camera model of the ideal optical system can be obtained with (12): 

$K_{f}$, the Scheimfplug camera imaging matrix, is

(12) $$\left(\begin{array}{ccc} f\cos\theta d_{x} & -f\sin\varphi \sin\theta d_{y} & -f\left(\cos\theta d_{x}x_{0}-\sin\varphi \sin\theta d_{y}y_{0}\right)\\ 0 & f\cos\varphi d_{y} & -f\cos\varphi d_{y}y_{0}\\ \left(e+f\right)\sin\theta d_{x} & \left(e+f\right)\sin\varphi \cos\theta d_{y} & \left(e+f\right)\left(\sin\theta d_{x}x_{0}-\sin\varphi \cos\theta d_{y}y_{0}+z_{0}\right)+ef \end{array}\right)$$

In the imaging matrix represented by (12), the parameters of the thick-lens hypothesis are in the third row, that is, they have a direct impact on the solution of $z_{c}$, but in fact, due to the coefficients in (11), the thick-lens hypothesis has a certain effect on the three coordinates.

Since the experimental objects are all rotating bodies, the scanning motion should use a rotary motion with no translational motion.

So ${\vec{t}}_{i}=\vec{0}$ in (13) and $R_{i}=\left(\begin{array}{ccc} \cos\left(i\Delta \omega \right) & -\sin\left(i\Delta \omega \right) & 0\\ \sin\left(i\Delta \omega \right) & \cos\left(i\Delta \omega \right) & 0\\ 0 & 0 & 1 \end{array}\right)$, where Δω is the angle of rotation of the object around the $z_{m}$-axis between measurements.

Finally, the linear-structured-light model is inferred by substituting $K_{f}$ into (4).

(13) $${\left(\begin{array}{ccc} x_{m} & y_{m} & z_{m} \end{array}\right)}^{T}=R_{i}\left(R_{c2m}K_{f}{\left(\begin{array}{ccc} x_{u} & y_{u} & 1 \end{array}\right)}^{T}/\left(\left(\begin{array}{ccc} A & B & C \end{array}\right)K_{f}{\left(\begin{array}{ccc} x_{u} & y_{u} & 1 \end{array}\right)}^{T}\right)+{\vec{t}}_{c2m}\right)$$

The above Equation (13) is the complete structured-light measurement model, from pixel-point coordinates to reconstructed object-point coordinates.

### 2.4. Linear-Structured-Light Model Calibration Step

In the linear-structured-light model (13), calibration is required to determine the values of the two parts of the matrix, $K_{f}$, the plane vector $\left(\begin{array}{ccc} A & B & C \end{array}\right)$, the rotation matrix $R_{c2m}$, and the translation vector ${\vec{t}}_{c2m}$, separately.

Considering that the parameters inside the camera are difficult to obtain, the $K_{f}$ calibration method consists of three steps, as follows: Step 1: a checkerboard calibration board with 15 positions is placed on the working range. Step 2: initial values are obtained from the nominal values of the device. f is the focal length of the lens, $d_{x}$ and $d_{y}$ are the size of the CCD pixel, $x_{0}$ and $y_{0}$ are the center of the CCD, φ and θ are the tilt angles of the Scheimfplug adapter, and e is set to 0 because it is theoretically very small. Step 3: the proposed model is validated using the L-M optimization algorithm, and the optimization principle is to minimize the reprojection error.

The calibration process of the plane vector $\left(\begin{array}{ccc} A & B & C \end{array}\right)$ can be performed by making the light stripe irradiate on the calibration plate plane and calculating the 3D coordinates of the light stripe in the camera coordinate system according to the camera calibration results. Since the light stripe is also on the laser plane, several different 3D light stripes can be fitted to the optical plane coefficients.

The rotation matrix, $R_{c2m}$, and translation vector, ${\vec{t}}_{c2m}$, are optimized with the ring gauge. The 3D coordinates of the light bar are reconstructed from the calibrated camera and light plane parameters by rotating the measured standard ring gauge. The obtained coordinates are subjected to a rigid body transformation to obtain a reconstructed model of the ring gauge. The evaluation parameter is set as the difference between the radius of the reconstructed cylinder and the nominal radius of the ring gauge. The optimization object is the rotation matrix and translation vector. The L-M (Levenberg–Marquardt) algorithm is applied to obtain the optimized parameters and realize the calibration.

## 3. Simulation and Experiment

### 3.1. Simulation

**Figure 3:**
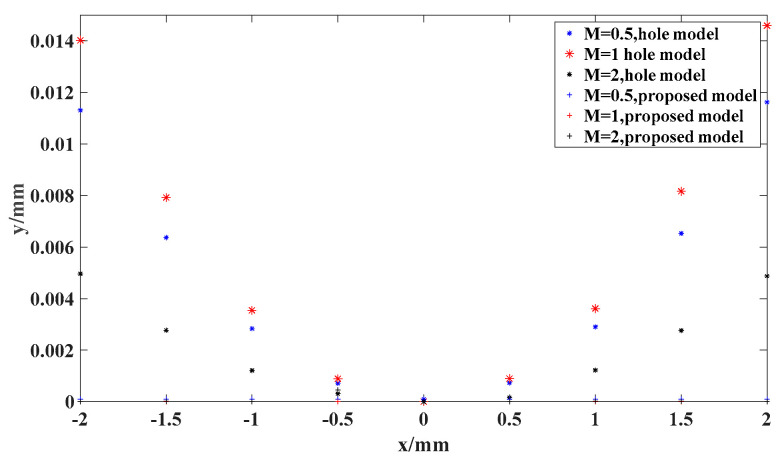
Simulation results for both models.

In this section, we discuss the numerical simulations that were conducted to test the performance of the proposed thick-lens hypothesis in a Scheimfplug camera. The simulation conditions were the solutions of the object plane coordinates from the image plane coordinates at $\beta_{m}=0.5,1,2$, and the solutions were compared with the position of the imaging point calculated by the law of refraction. The simulation results in Figure 3 show that the aperture model led to increased computational deviation as the image plane coordinates kept moving away from the center point at different magnifications. In contrast, the accuracy of the proposed thick-lens model was maintained even with the change in image plane position.

### 3.2. Experimental Section 

This experimental part includes four sections. Section 3.2.1 describes the experimental structure, including the equipment and structure of the line-structured-light measurement system built in the experiment. Section 3.2.2 describes the calibration of the line-structured-light measurement model, including the imaging matrix, light plane, rotation matrix, and translation vector. Section 3.2.3 describes how to measure the standard measurement block with the measuring device, use two models to solve the measurement results, and compare the mean value and standard deviation of the two results. Section 3.2.4 describes the use of measuring devices to measure a real bearing, proving that the proposed model has a good effect in practical industrial measurement.

#### 3.2.1. Experimental Structure

**Figure 4:**
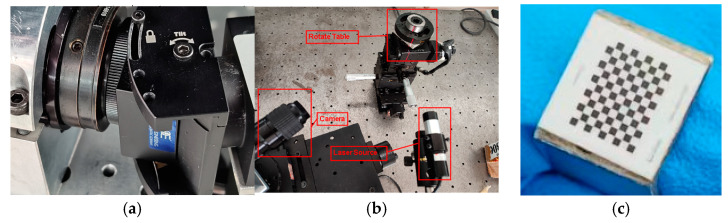
Experimental equipment: (**a**). Scheimfplug transfer interface. (**b**). Linear-structured-light measurement system. (**c**). Checkboard calibration target.

A zoom 6000 camera from Navitar (Rochester, NY, USA) and Scheimpflug adapter were used to build a Scheimpflug camera. The CCD was GRAS-20S4M-C, 1/1.8inch, with 1200 × 1600 pixels, and the pixel size was 4.4 μm, as in Figure 4a. The lens tilt of the Scheimfplug adapter ranged from 0° to 17°. 

A linear-structured-light system was designed containing a Scheimfplug camera and a linear laser, as in Figure 4b.

#### 3.2.2. Line-Structured-Light Measurement System Calibration Experiment

The calibration plates in Figure 4c were used to compare the two camera models. Experiments were performed on both the Scheimfplug camera and the regular camera at both ×1.25 and ×1.5 magnifications, and the relevant calibration results are shown in Table 1 and Table 2. In Table 1, each parameter corresponds to the matrix $K_{f}$ in (13), whereas in Table 2, each parameter corresponds to the matrix $K_{h}$ in (14).

(14) $$K_{h}=\left(\begin{array}{ccc} \alpha & \gamma & x_{0}\\ 0 & \beta & y_{0}\\ 0 & 0 & 1 \end{array}\right)$$

The above Equation (14) is the imaging matrix of the pinhole model, which represents a special linear transformation, and the matrix is an upper triangular matrix. The units of the reprojection errors in the two tables are not the same due to the different objects of calibration optimization; with pixels being applied to the traditional small-aperture model and μm being used for the proposed model, it is difficult to analyze the two in terms of how good they are in terms of the results of the camera calibration.

The Scheimfplug camera with a magnification of ×1.5 in Table 1 was selected to form a measurement system with the laser and the optical plane was calibrated, and the results were $10^{-4}\left(\begin{array}{ccc} 8.6089 & 3.6205 & 31.749 \end{array}\right)$.

**Figure 5:**
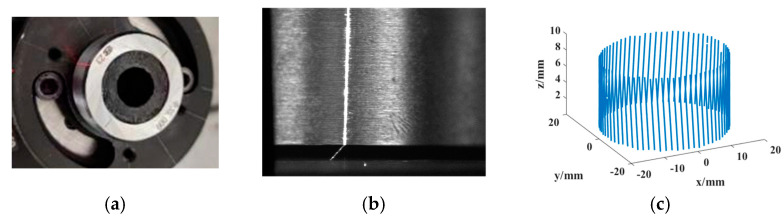
Calibration ring gauge and calibration image: (**a**). Ring gauge. (**b**). Ring gauge light stripe image. (**c**). Three-dimensional reconstruction of environmental regulations.

The $R_{c2m}$ and ${\vec{t}}_{c2m}$ of the measurement system were calibrated using a ring gauge with a radius of 17.500 mm. The system was calibrated against the reconstructed 3D model by rotating the ring gauge once on a rotary table, as shown in Figure 5a. The ring gauge optical strip image is shown in Figure 5b. and the 3D reconstructed cylinder is shown in Figure 5c. The calibration results were as follows:

$$R_{c2m}=\left(\begin{array}{ccc} 0.41199 & 0.036022 & -0.91047\\ 0.082152 & 0.99368 & 0.076488\\ 0.90748 & -0.10631 & 0.40643 \end{array}\right),{\vec{t}}_{c2m}=\left(\begin{array}{c} -10.023\\ -300.06\\ 11.231 \end{array}\right)$$

The deviation of the reconstructed cylindrical radius from the standard ring gauge in Figure 5 is 1.2 μm.

#### 3.2.3. Single-Measurement Model Validation Experiment

**Figure 6:**
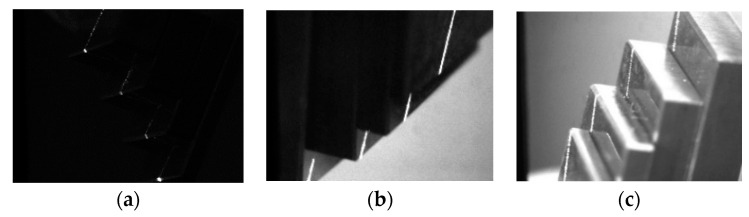
Measurement images of measuring blocks under different ambient lights: (**a**). Dark. (**b**). Low light. (**c**). Bright.

**Figure 7:**
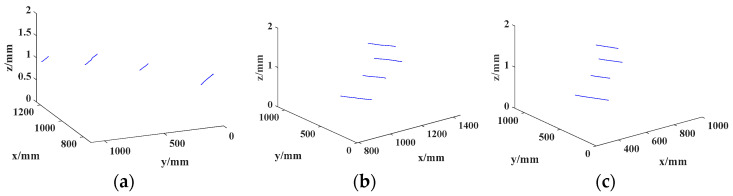
Three-dimensional coordinates of the light strip corresponding to the results in Figure 6: (**a**) Corresponding to Figure 6a. (**b**) Corresponding to Figure 6b. (**c**) Corresponding to Figure 6c.

Three measurement blocks of 1 mm, 1.5 mm, and 2 mm, respectively, were stacked to form a stepped block as a measurement object to verify the model’s validity. The Scheimpflug camera captured an image of the target, as shown in Figure 6. After image processing and 3D reconstruction, the position of the stripe in the 3D space, i.e., the single measurement of the block, was obtained. Then, the measurements were compared with the real values. Since the target was randomly moved to different positions, enough results were obtained to evaluate our measurement system, but it was, therefore, impossible to obtain specific measurements, only the height ratio of the stepped blocks. The 3D results are shown in Figure 7 and listed in Table 3.

The standard values of the height ratio were 1, 1.5, and 2. Correspondingly, the relative deviations were 0, 0.6%, and 0.8%, respectively, when using the proposed model, while the pinhole model’s relative deviations were 0, 1.3%, and 3.2%. The proposed deviations of the model were reduced by 0.7% except for the first item because the first was necessarily 0 and not worth discussing. The evaluation results were sufficiently accurate as the blocks’ uncertainty was 0.02 µm. The experiments showed that the proposed thick-lens model had a higher accuracy and lower standard deviation than the traditional small-hole model.

#### 3.2.4. Measurement Experiments of Real Objects

**Figure 8:**
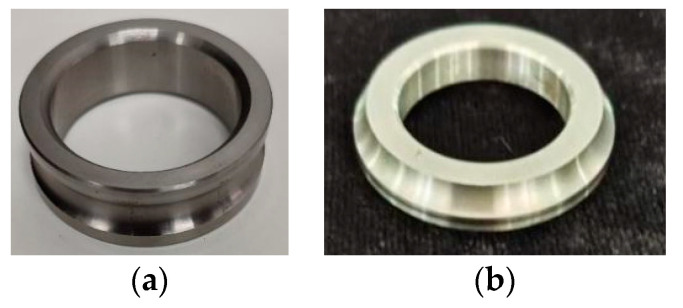
Bearing inner ring: (**a**) Big bearing inner ring. (**b**) Half of small bearing inner-half ring.

**Figure 9:**
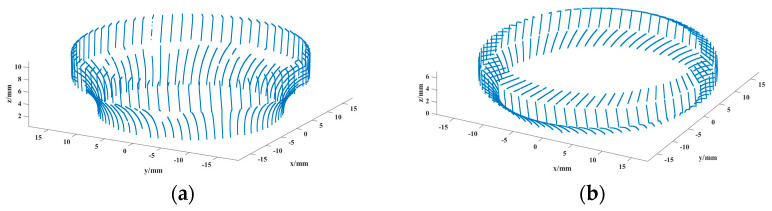
Bearing reconstruction results: (**a**) Big bearing inner ring. (**b**) Half of small bearing inner-half ring.

**Figure 10:**
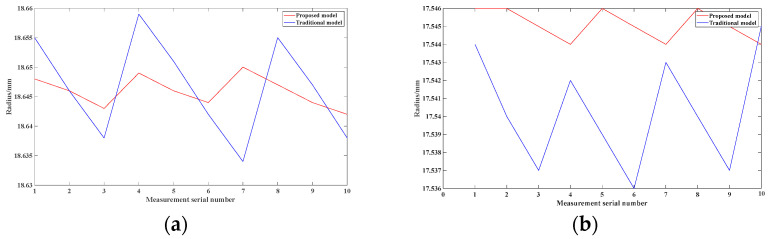
Bearing measurement results: (**a**) Large bearing results. (**b**) Small bearing results.

The radii of the inner rings of the two bearings were measured using the parameters obtained from the calibration; the measurement objects are shown in Figure 8, the 3D measurement results are shown in Figure 9, and the results of 10 measurements of the radii are shown in Figure 10.

The bearing radii calculated using the proposed model are 18.646 mm and 17.545 mm with standard deviations of 0.003 mm and 0.001 mm, respectively, while the results of the conventional model are 18.646 mm and 17.540 mm with standard deviations of 0.009 mm and 0.003 mm, respectively. The standard deviation of the proposed model is significantly lower. The effectiveness of the structured-light measurement system based on the thick-lens model is verified.

## 4. Discussion

The introduction of a Scheimpflug camera thick-lens imaging model in this study significantly enhances the precision of structured-light measurement. This model is rooted in the principles of geometrical optics and the thick-lens hypothesis, making it a more accurate representation of real-world measurement systems. The experimental outcomes have demonstrated a remarkable reduction in the standard deviation by 66% when compared to the pinhole model, validating the effectiveness of the proposed model in improving measurement accuracy.

The model’s derivation from fundamental optical principles allows it to more closely mimic the behavior of actual cameras, which typically have a non-negligible lens thickness. This approach addresses the limitations of traditional models that assume a negligible lens thickness, leading to inaccuracies in measurement. The practical utility of this model is evident as it can be potentially applied to other structured-light measurement systems where lens thickness plays a significant role. This adaptability makes the model a valuable tool for improving the performance of various imaging systems.

Moreover, the model’s practicability is highlighted by its simplicity and effectiveness. Unlike many complex models that require intricate calibration processes, this model offers a more straightforward approach to achieving high-precision measurements. This characteristic is particularly beneficial in industrial applications with critical ease of use and reliability.

The results of this study open avenues for further research and development. Future work will concentrate on simplifying the calibration algorithm of the model. This endeavor aims to streamline the process of obtaining accurate camera and motion parameters, thereby enhancing the overall measurement accuracy. The focus will be on developing algorithms that are precise and easy to implement, making the technology more accessible and user-friendly.

## 5. Conclusions

In conclusion, the Scheimpflug camera thick-lens imaging model proposed in this study offers a significant advancement in structured-light measurement. The model’s foundation in geometrical optics and the thick-lens hypothesis provides a more realistic and accurate representation of camera systems, leading to improved measurement precision. The experimental results confirm a 66% reduction in the standard deviation compared to the traditional pinhole model, showcasing the model’s superior performance.

The proposed model’s practicality and adaptability make it a promising candidate for enhancing the performance of other structured-light measurement systems. Its straightforward calibration process and robust performance underpin its potential for widespread applications in various industrial settings.

Looking ahead, the focus will be on refining the model’s calibration algorithm to achieve even greater accuracy in camera and motion parameter determination. This will further enhance the model’s utility and precision, solidifying its role as a key technology in the field of structured-light measurement. The ongoing commitment to improving the model’s calibration and measurement capabilities will ensure its continued relevance and effectiveness in addressing the challenges of high-precision imaging systems.

## Figures and Tables

**Table 1:** Calibration results for models in this paper.

Scheimfplug Camera			
Magnification		1.25	1.5
$\mathrm{Matrix}\;K_{f}$ parameter	f	100.01	97.004
	$x_{0}$	807.88	796.21
	$y_{0}$	598.82	593.99
	φ	−0.34243	2.8718
	θ	13.749	13.307
	e	−0.97048	−2.4125
	$z_{0}$	256.31	249.47
	$d_{x}$	0.0049	0.0049
	$d_{y}$	0.0051	0.0048
Reprojection error (μm)		1.6	1.3
General camera model			
Magnification		1.25	1.5
$\mathrm{Matrix}\;K_{f}$ parameter	f	99.749	86.920
	$x_{0}$	801.78	799.64
	$y_{0}$	596.79	601.90
	φ	−5.7397	0.46466
	θ	5.8230	0.43254
	e	−2.1826	−2.6541
	$z_{0}$	248.92	250.85
	$d_{x}$	0.0049	0.0049
	$d_{y}$	0.0049	0.0049
Reprojection error (μm)		1.6	1.7

**Table 2:** Calibration results of small-hole model.

Scheimfplug Camera			
Magnification		1.25	1.5
Matrix K parameter	α	36,803	39,326
	β	32,676	36,572
	γ	1411.3	256.63
	$x_{0}$	−4042.5	−1818.3
	$y_{0}$	−490.96	504.58
Reprojection error (pixel)		0.68	0.24
General camera model			
Magnification		1.25	1.5
Matrix K parameter	α	38,821	37,285
	β	38,792	37,241
	γ	−37.424	−18.943
	$x_{0}$	481.41	557.03
	$y_{0}$	649.06	587.87
Reprojection error (pixel)		0.21	0.18

**Table 3:** Gauge measurement results.

	Standard Value	1st Measure	2nd Measure	3rd Measure	Height Ratio
New model	1.000 ± 0.0002	1.022	1.014	1.036	1
	1.500 ± 0.0002	1.521	1.510	1.548	1.491
	2.000 ± 0.0002	2.020	2.026	2.050	1.984
Traditional model	1.000 ± 0.0002	1.008	0.999	1.018	1
	1.500 ± 0.0002	1.534	1.516	1.546	1.519
	2.000 ± 0.0002	2.077	2.078	2.089	2.064

## Data Availability

Data is contained within the article.
